# Complete laparoscopic and Da Vinci robot esophagogastric anastomosis double muscle flap plasty for radical resection of proximal gastric cancer

**DOI:** 10.3389/fonc.2024.1395549

**Published:** 2024-06-04

**Authors:** Dong Yang, Yuanlin Liu, Xiangyu Meng, Xing Xu, Chao Wang, Meng Zhang, Tao Zhang

**Affiliations:** Department of Gastrosurgery, Liaoning Cancer Hospital & Institute, Shenyang, China

**Keywords:** stomach neoplasms, upper stomach, radical resection of proximal gastric cancer, kamikawa anastomosis, complications, laparoscopy, Da Vinci robot

## Abstract

**Objective:**

To investigate the application value of complete laparoscopy and Da Vinci robot esophagogastric anastomosis double muscle flap plasty in radical resection of proximal gastric cancer.

**Method:**

A retrospective descriptive study was used. The clinicopathological data of 35 patients undergoing radical operation for proximal gastric cancer admitted to Liaoning Cancer Hospital from January 2020 to December 2023 were collected. Variables evaluated: 1. Transoperative,2. Postoperative, 3. Follow-up. In relation to follow-up, esophageal disease status reflux, anastomosis, nutritional status score, serum hemoglobin, tumor recurrence, and metastasis were investigated. The trans and postoperative variables were obtained from the clinical records and the patients were followed up in outpatient department and by telephone.

**Result:**

Among the 35 patients, 17 underwent robotic surgery and 18 underwent laparoscopic surgery. There were 29 males and 6 females. 1) Transoperative: Robotic surgery: The operation time was (305.59 ± 22.07) min, the esophagogastric anastomosis double muscle flap plasty time was (149.76 ± 14.91) min, the average number of lymph nodes cleared was 30, and the average intraoperative blood loss was 30 ml. Laparoscopic surgery: The mean operation time was 305.17 ± 26.92min, the operation time of esophagogastric anastomosis double muscle flap was (194.06 ± 22.52) min, the average number of lymph nodes cleared was 24, and the average intraoperative blood loss was 52.5 ml. 2) Postoperative: Robotic surgery: the average time for patients to have their first postoperative anal emission was 3 days, the average time to first postoperative feeding was 4 days, and the average length of hospitalization after surgery was 8 days. Laparoscopic surgery: the average time for patients to have their first postoperative anal emission was 5 days, the average time to first postoperative feeding was 6 days, the average length of hospitalization after surgery was 10 days. 3) Follow-up: The follow-up time ranged from 1 to 42 months, with a median follow-up time of 24 months.

**Conclusion:**

Complete Da Vinci robot and laparoscopic esophagogastric anastomosis double muscle flap plasty for radical resection of proximal gastric cancer can minimize surgical incision, reduce abdominal exposure, accelerate postoperative recovery of patients, and effectively prevent reflux esophagitis and maintain good hemoglobin concentration and nutritional status. The advantages of robotic surgery is less intraoperative bleeding and faster post-surgical recovery, but it is relatively more expensive.

## 1 Introduction

In recent years, the incidence of upper gastric cancer and esophagogastric junction tumor has been increasing year by year. The incidence of proximal gastric cancer shows a steady increasing trend (1). In order to preserve part of the gastric function and reduce nutritional complications, these patients have undergone multiple proximal gastrectomy. However, no standard method for digestive tract reconstruction after proximal gastrectomy has been established, mainly due to the postoperative gastroesophageal reflux problem. In 2016, Kuroda et al. (2) reported a laparoscope assisted esophagogastric anastomosis double muscle flap plasty first performed by Japanese scholar Muraoka after proximal gastrectomy. This anastomosis method is direct esophagogastric anastomosis, which is similar to reconstruction of cardiac after anastomosis. This operation has good anti-reflux effect, high postoperative life quality, anastomosis wrapped by sarcoplasmic layer and low incidence of anastomotic fistula. However, the double-flap technique requires complex suture and is difficult to perform laparoscopy. In recent years, surgical robots have emerged to overcome some disadvantages of laparoscopic surgery (3–6). The advantages of the robot, such as multi-degree of freedom rotatable wrist device, high-definition enlarged 3D field of view and filtered hand tremor, make it easy to suture, which greatly reduces the operation difficulty of complete endoscopic digestive tract reconstruction suture, and manual suture can reduce related equipment costs and overall costs (7). In 2017, Shibasaki et al. reported for the first time the application of robot-assisted esophagogastric anastomosis double muscle flap plasty in proximal gastrectomy (8). However, complete robotic proximal subtotal gastrectomy, esophagogastric anastomosis and double muscle flap plasty have not been reported. We found that the da Vinci robot and laparoscopic esophagogastric anastomosis with double muscle flap plasty for radical resection of proximal gastric cancer minimizes surgical incisions, reduces abdominal exposure, accelerates patients’ postoperative recovery, effectively prevents reflux esophagitis, and maintains good hemoglobin concentration and nutritional status. The da Vinci robot is superior in controlling intraoperative bleeding and postoperative recovery, and laparoscopic surgery is less expensive. The objective was to investigate the value of application of complete laparoscopy and Da Vinci Robot esophagogastric anastomosis plasty with double muscle flap in radical resection of proximal gastric cancer.

## 2 Materials and methods

### 2.1 General information

This is a retrospective, descriptive and comparative study. The clinicopathological data of 35 patients undergoing radical resection for proximal gastric cancer were collected. The tumor sites of 35 patients were all upper gastric cancer, and the preoperative pathological examination results showed adenocarcinoma, and the preoperative clinical stage was cT1a-bN0M0 (9). This study was approved by the Medical Ethics Committee of Liaoning Cancer Hospital. Informed consent forms are signed by patients and their families (**Table 1**).

**Table 1:** General information of patients.

Characteristics	Laparoscopic surgery	Da Vinci robotic-assisted	P value
n	18	17	
Age, mean ± sd	62.56 ± 6.17	64.05 ± 4.96	0.434
Gender, n (%)			1.000
M	15 (83.3%)	14 (82.4%)	
F	3 (16.7%)	3 (17.6%)	
BMI, mean ± sd	23.63 ± 2.59	23.11 ± 2.65	0.564
Stage, n (%)			1.000
I	15 (83.3%)	15 (88.2%)	
II	3 (16.7%)	2 (11.8%)	

### 2.2 Inclusion criteria and exclusion criteria

Inclusion criteria: 1) Preoperative gastroscopic biopsy confirmed adenocarcinoma of upper stomach. 2) Preoperative CT, MRI, ultrasound and other imaging examinations did not show distant organ metastasis. 3) Complete robotic and laparoscopic radical proximal subtotal gastrectomy and esophagogastric anastomosis double muscle flap plasty were performed. 4) Complete follow-up data. 5) No history of abdominal surgery. 6) No blood system diseases.

Exclusion criteria: 1) Tumor involvement in the lower esophagus. 2) No radical surgery was performed. 3) Complicated with severe cardiopulmonary dysfunction, cerebrovascular disease, liver and kidney insufficiency. 4) Preoperative radiotherapy, chemotherapy, immunotherapy and other treatment means. 5) Preoperative examination combined with other malignant tumors or a history of malignant tumors. 6) Refusing to sign the informed consent for surgery. 7) The follow-up data were incomplete.

### 2.3 Surgical methods

**Figure 1:**
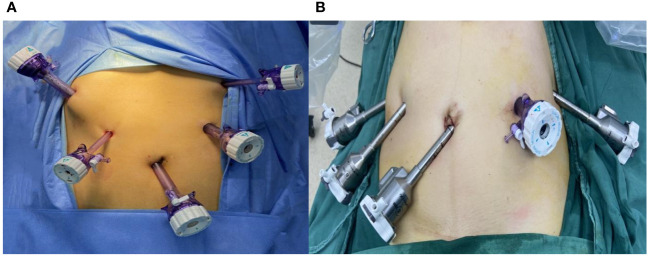
**(A)** Trocar position in laparoscopic surgery **(B)** Trocar position in robotic surgery.

(1) Patient position and anesthesia: under general anesthesia, the patient is in the inverted Trendelenburg position and with a right inclination of 15 degrees.
(2) Tumor location: After laparoscopic exploration of the entire abdomen, a gastroscopy was performed to confirm the location of the tumor, and the surgeon used sutures to mark it.
(3) Trocar location: In the laparoscopic technique, the 5-trocar method was adopted. Connection of robotic surgical system: 8 mm Trocar was inserted 1 cm below the umbilicus to establish pneumoperitoneum; The 8 mm Trocar was inserted 2 cm below the costal margin of the right anterior axillary line, at the flat umbilical line of the right midclavicular line, and 2 cm below the costal margin of the left anterior axillary line, respectively, as the operating holes of robot arms No. 1, No. 2 and No. 4. The No. 1 mechanical arm is equipped with a non-damaging grip to assist in exposure, the No. 2 mechanical arm is equipped with a bipolar electrocoagulation clamp, the No. 3 mechanical arm is installed with a camera, the No. 4 mechanical arm is installed with an ultrasonic knife system and unipolar electrocoagulation scissors, and the needle is installed when gastrointestinal anastomotic and stump embedding. The auxiliary hole was punctured using a 12 mm Trocar at the flat umbilicus of the left midclavicle line. The robotic surgical system enters the machine vertically from the left side of the patient, rotates the mechanical lifting arm so that it is facing the patient’s head side, and successively installs the mechanical arm and sets the human lens and operating instruments. See **Figure 1**.

### 2.4 Proximal gastrectomy and lymph node dissection

**Figure 2:**
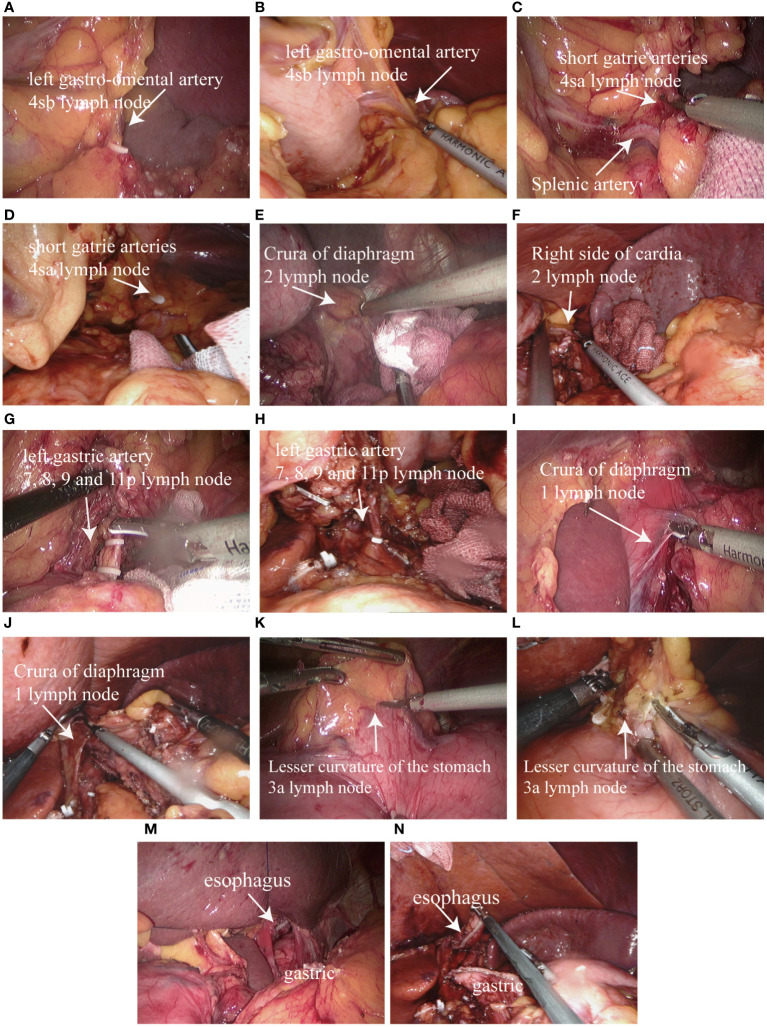
Proximal subtotal gastrectomy and lymph node dissection: **(A)** Dissecting the left gastro-omental artery and group 4sb lymph node dissection in laparoscopic surgery; **(B)** Dissecting the left gastro-omental artery and group 4sb lymph node dissection in Da Vinci robotic surgery; **(C)** Dissecting the short gatrie arteries and group 4sa lymph node dissection in laparoscopic surgery; **(D)** Dissecting the short gatrie arteries and group 4sa lymph node dissection in Da Vinci robotic surgery; **(E)** Group 2 lymph node dissection in laparoscopic surgery; **(F)** Group 2 lymph node dissection in Da Vinci robotic surgery; **(G)** Dissecting the left gastric artery and group 7, 8, 9 and 11p lymph node dissection in laparoscopic surgery; **(H)** Dissecting the left gastric artery and group 7, 8, 9 and 11p lymph node dissection in Da Vinci robotic surgery; **(I)** Group 1 lymph node dissection in laparoscopic surgery; **(J)** Group 1 lymph node dissection in Da Vinci robotic surgery; **(K)** Group 3a lymph node dissection in laparoscopic surgery; **(L)** Group 3a lymph node dissection in Da Vinci robotic surgery; **(M)** Separated from the esophagus, separated from the stomach in laparoscopic surgery; **(N)** Separated from the esophagus, separated from the stomach in Da Vinci robotic surgery.

After routine exploration under laparoscopic and robotic lens to exclude abdominal metastasis and other lesions, the hepatogastric ligament was cut along the lower margin of the liver by ultrasonic knife, gradually separated upward to the right side of the cardiac, cut part of the right diaphragmatic foot, and suspended the liver through the abdominal wall. Lymph node dissection was performed in accordance with the 5th Japanese Guidelines for the Treatment of Gastric Cancer (10). The omentum and part of the anterior lobe of the transverse mesocolon were separated from right to left along the vascularized area between the transverse colon and the omentum, and the left arteriovenous gastroomentum was severed by vascular clamp at the root. Lymph nodes in group 4sb were removed (**Figures 2A, B**). The short gastric vessels were removed upward and lymph nodes in group 4sa were cleared (**Figures 2C, D**). Lymph nodes in group 2 were removed from the right side of the cardia (**Figures 2E, F**). The root was removed from the greater omentum under the right arterial arch of the gastroomentum to the arteriovenous junction of the left and right gastroomentum. The gastropancreatic fold was separated to expose the upper margin of pancreas. The common hepatic artery, the proximal end of the splenic artery and the root of the left gastric artery were separated and the left gastric artery was removed at the root. Lymph nodes in groups 7, 8, 9 and 11p were cleaned (**Figures 2G, H**). The lesser omentum was separated from the left side of the right gastric artery along the superior margin of the pylorus, and the lymph nodes in group 1 (**Figures 2I, J**) and Group 3a (**Figures 2K, L**) were cleared, and the esophagus was fully freed and the lateral vagus nerve trunk was severed. Under the Da Vinci robot and laparoscope, the esophagus and stomach were severed by a linear cutting and closing device (**Figures 2M, N**). The stomach specimen was placed in the specimen bag, the pneumoperitoneus was closed, the Da Vinci robot and the laparoscopic mechanical arm were removed, and the small incision around the umbilicus was about 3.5cm long. The specimens were removed to confirm the proximal and distal incisal margin of the tumor.

### 2.5 Esophagogastric anastomosis double muscle flap plasty

**Figure 3:**
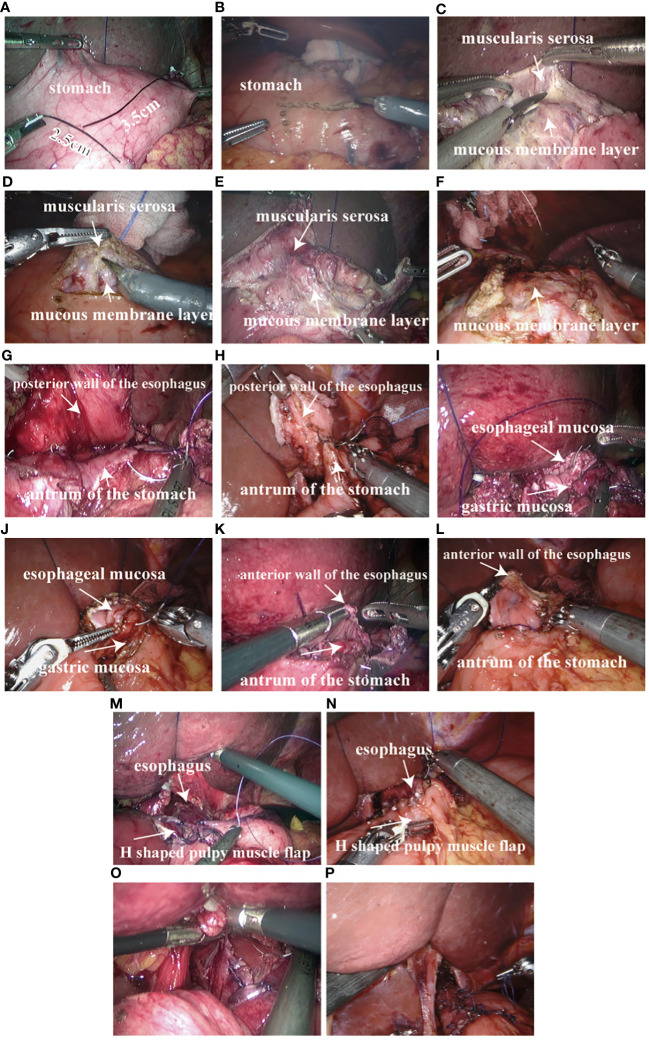
Proximal gastrectomy Esophagogastric anastomosis double muscle flap plasty: **(A)** 3~4 cm away from the proximal stump of the stomach marked with an “H” shape, 2.5cm in width and 3.5cm in upper and lower spacing in laparoscopic surgery; **(B)** 3~4 cm away from the proximal stump of the stomach marked with an “H” shape, 2.5cm in width and 3.5cm in upper and lower spacing in Da Vinci robotic surgery; **(C)** double muscle flap made by laparoscopic surgery; **(D)** double muscle flap made by Da Vinci robotic surgery; **(E)** double muscle flap made by laparoscopic surgery; **(F)** double muscle flap made by Da Vinci robotic surgery; **(G)** The posterior wall of the esophagus and the stomach wall “H” shaped muscle flap across the upper side of the barb line continuous suture in laparoscopic surgery; **(H)** The posterior wall of the esophagus and the stomach wall “H” shaped muscle flap across the upper side of the barb line continuous suture in Da Vinci robotic surgery; **(I)** The whole posterior wall of the esophagus and the “H” -shaped transverse mucosa and submucosa of the residual stomach were sutured with barb line continuously in laparoscopic surgery; **(J)** The whole posterior wall of the esophagus and the “H” -shaped transverse mucosa and submucosa of the residual stomach were sutured with barb line continuously in Da Vinci robotic surgery; **(K)** The whole anterior wall of the broken end of the esophagus and the whole lower anal side of the residual stomach in the shape of “H” were sutured with barb line continuously in laparoscopic surgery; **(L)** The whole anterior wall of the broken end of the esophagus and the whole lower anal side of the residual stomach in the shape of “H” were sutured with barb line continuously in Da Vinci robotic surgery; **(M)** muscle layer reinforcement in laparoscopic surgery; **(N)** muscle layer reinforcement in Da Vinci robotic surgery; **(O)** The “H” shaped serosal muscle flap of the anterior wall of the stomach was used for “Y” shaped intermittent suture wrap around the anastomosis in laparoscopic surgery; **(P)** The “H” shaped serosal muscle flap of the anterior wall of the stomach was used for “Y” shaped intermittent suture wrap around the anastomosis in Da Vinci robotic surgery.

The pneumoperitoneum was re-established, the Da Vinci robot and the laparoscopic mechanical arm were connected, and the pneumoperitoneum pressure was maintained at 10~12 mmHg (1 mmHg= 0.133kPa). The complete Da Vinci robot and the laparoscopic unipolar electrocoagulative scissors on the residual stomach marked an “H” shape, which was 3 ~ 4 cm away from the top of the residual stomach and about 2.5cm wide. The upper and lower spacing is about 3.5cm (**Figures 3A, B**). Serosal membrane and gastric muscle layer were separated along the “H” shaped marking line to make serosal muscle flap. Unipolar electrocoagulating scissors cut the serosal layer and muscle layer on both sides of the “H” shape and in the middle of the longitudinal line, and separated the serosal muscle flap from the middle to the sides along the gap between the submucosa and muscle layer without injury and the appropriate tension of the assistant pulling (**Figures 3C–F**). The mucosa was cut about 2.5cm below the transverse “H” shape for subsequent esophagogastric anastomosis. The posterior wall of the esophagus was pulled 4 cm away from the broken end of the esophagus, and the “H” shaped serosal muscle flap of the stomach wall was continuously sutured across the upper barb line (**Figures 3G, H**). Fix the severed end of the esophagus and the residual stomach. The closed end of the esophagus was cut open and the esophageal stump stump was anastomosed. The whole posterior wall of the esophageal stump and the cephalic mucosa and submucosa of the stump were continuously sutured with barbs (**Figures 3I, J**). The whole anterior wall of the broken end of the esophagus and the whole lower anal side of the residual stomach in an “H” shape were continuously sutured with barbs (**Figures 3K, L**), and the sarcomuscular layer was reinforced (**Figures 3M, N**). The “H” -shaped serosal muscle flap of the anterior gastric wall was used as a “Y” -shaped intermittent suture wrap around the anastomosis (**Figures 3O, P**). Gastroscopy was performed during the operation to determine whether the esophageal residual gastric anastomosis was complete, unobstructed and whether there was bleeding. After the wound was completely hemostatic, the abdominal cavity was fully rinsed with distilled water, and a drainage tube was placed behind the Wentz foramen or esophagogastric anastomosis.

### 2.6 Observation indicators and evaluation criteria

Outcome measures: 1) Transoperative: operation completion, operation time, number of lymph node dissection, intraoperative blood loss, incision length, and intraoperative esophageal margin. 2) Postoperative: time of first postoperative anal exhaust, time of first postoperative feeding, time of postoperative hospitalization, treatment cost, postoperative pathological stage, postoperative pathological examination, and complications. 3) Follow-up: the number of patients who were followed up, follow-up time, tumor recurrence and metastasis, postoperative anastomosis, occurrence of reflux esophagitis, postoperative nutritional status and serum hemoglobin.

Evaluation criteria: Pathological staging was performed according to TNM staging criteria of the American Cancer Federation (AJCC) (5). Postoperative nutritional status was assessed by BMI, nutritional risk screening score (NRS2002 score), and patient subjective global assessment score (PG-SGA score) (11–13).

### 2.7 Follow-up

The patients were followed up in outpatient department and by telephone. The status of esophageal reflux, anastomosis, nutritional status, serum hemoglobin, tumor recurrence and metastasis were investigated. The follow-up period ends in December 2023.

### 2.8 Statistical analysis

SPSS 19.0 statistical software was used for analysis. The measurement data of normal distribution is expressed as x ± s, the measurement data of skew distribution is expressed as M (range), and the counting data is expressed as absolute number.

## 3 Result

Among the 35 patients, 29 were males and 6 were females. The median age was 63 years, and the age range was 53-72 years.

### 3.1 Transoperative

Robotic surgery: The operation time was (305.59 ± 22.07) min, the esophagogastric anastomosis double muscle flap plasty time was (149.76 ± 14.91) min, the average number of lymph nodes cleared was 30, and the average intraoperative blood loss was 30 ml. The average incision length was 3.2 cm, and no malignant components were found in the intraoperative rapid frozen section of the esophageal margin. Laparoscopic surgery: The mean operation time was (305.17 ± 26.92) min, the operation time of esophagogastric anastomosis double muscle flap was (194.06 ± 22.52) min, the average number of lymph nodes cleared was 24, and the average intraoperative blood loss was 52.5 ml. The average incision length was 3.3 cm, and no malignant elements were found in frozen esophageal margins during operation. Comparison of esophagogastric anastomosis double muscle flap angioplasty time, blood loss and lymph node dissection between the two groups had statistical differences (P<0.05) (**Table 2**).

**Table 2:** Surgical data of patients.

Characteristics	Laparoscopic surgery	DaVinci robotic-assisted	P value
n	18	17	
Esophagogastric anastomosis double muscle flap plasty time, mean ± sd	194.06 ± 22.52	149.76 ± 14.91	< 0.001
Operation time(min), mean ± sd	305.17 ± 26.92	305.59 ± 22.07	0.960
Intraoperative blood loss(ml), median (IQR)	52.5 (50, 55)	30 (30, 35)	< 0.001
Incision length(cm), median (IQR)	3.3 (3.125, 3.5)	3.2 (3.1, 3.5)	0.519
Number of lymph nodes, median (IQR)	24 (22.25, 25)	30 (29, 31)	< 0.001

IQR, Interquartile Range.

### 3.2 Postoperative

The pathologic type of all patients was adenocarcinoma, with no malignant component seen at the proximal margin or distal margins.

**Figure 4:**
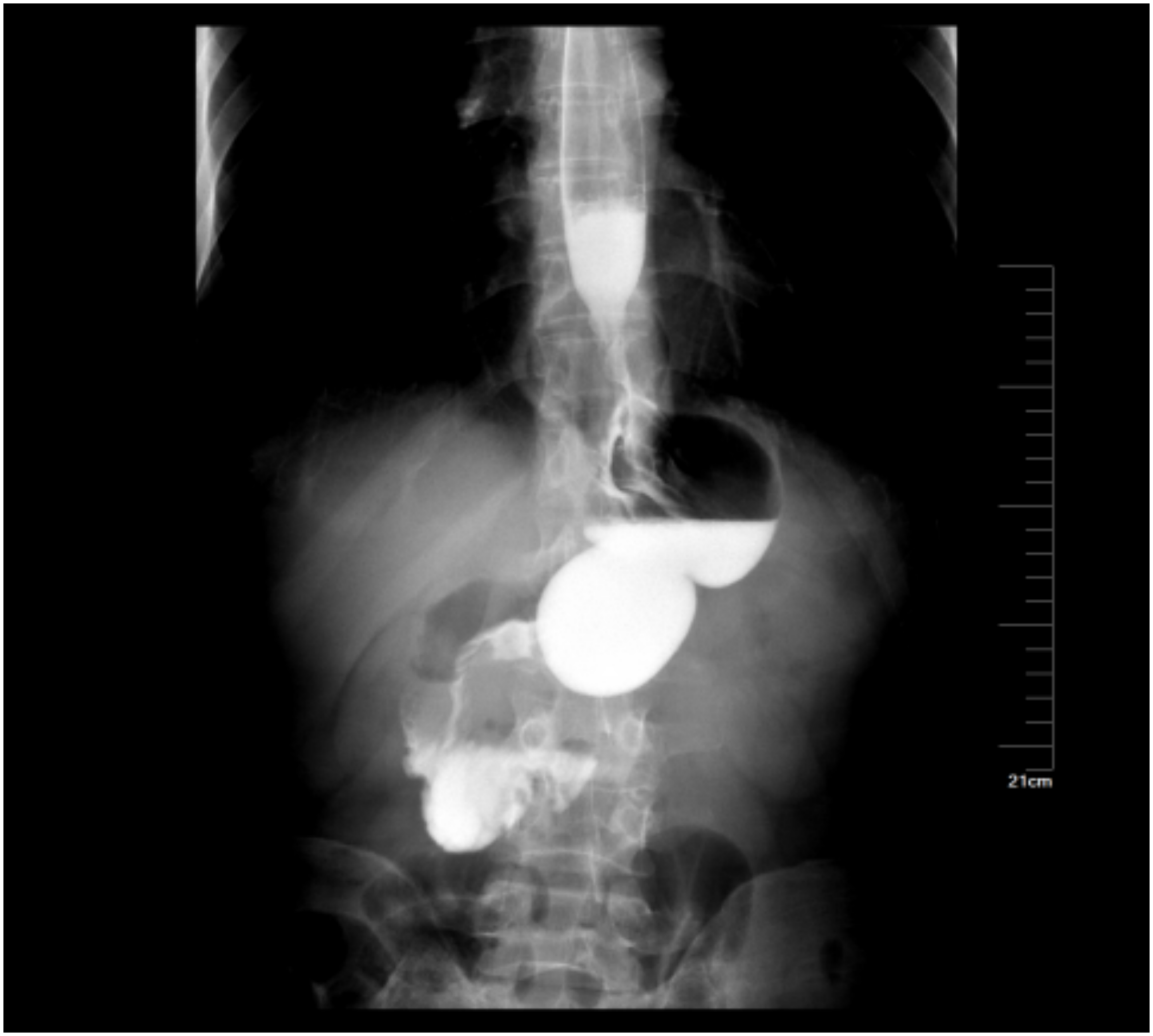
Postoperative upper gastrointestinal contrast study of the patient showed that the anastomosis was unobstructed.

None of the patients had severe complications, such as bleeding, anastomotic fistula and infection (**Figure 4**).

Robotic surgery: The average time for patients to have their first postoperative anal emission was 3 days, the average time to first postoperative feeding was 4 days, the average length of hospitalization after surgery was 8 days, and the treatment cost was (7.99 ± 0.37) ten thousand yuan. Laparoscopic surgery: The average time for patients to have their first postoperative anal emission was 5 days, the average time to first postoperative feeding was 6 days, the average length of hospitalization after surgery was 10 days, and the treatment cost was (5.79 ± 0.36) million yuan. The patient obtained good serum hemoglobin, nutritional status and quality of life (**Table 3**).

**Table 3:** Postoperative data of patients.

Characteristics	Laparoscopic surgery	DaVinci robotic-assisted	P value
n	18	17	
First anal exhaust time (day), median (IQR)	5 (5, 5.75)	3 (3, 4)	< 0.001
First postoperative feeding time (day), median (IQR)	6 (6, 6.75)	4 (4, 5)	< 0.001
Postoperative hospitalization time (day), median (IQR)	10 (9, 10)	8 (7, 9)	< 0.001
Treatment cost (ten thousand yuan), mean ± sd	5.79 ± 0.36	7.99 ± 0.37	< 0.001
Reflux esophagitis, n (%)			1.000
NO	17 (94.4%)	17 (100%)	
YES	1 (5.6%)	0 (0%)	
Nastomotic stenosis, n (%)			1.000
NO	17 (94.4%)	17 (100%)	
YES	1 (5.6%)	0 (0%)	
NRS2002 score, n (%)			0.505
1	8 (44.4%)	10 (58.8%)	
2	10 (55.6%)	7 (41.2%)	
PG-SGA score, n (%)			0.830
1	6 (33.3%)	7 (41.2%)	
2	7 (38.9%)	7 (41.2%)	
3	5 (27.8%)	3 (17.6%)	
QOL score, mean ± sd	45.78 ± 3.49	47.176 ± 4.00	0.278
Serum hemoglobin(g/L), median (IQR)	121 (117.25, 129.25)	123 (119, 133)	0.597
BMI, mean ± sd	23.63 ± 2.59	23.11 ± 2.65	0.564

IQR, Interquartile Range.

BMI, Body Mass Index.

QOL score, Quality of life.

### 3.3 Follow-up

**Figure 5:**
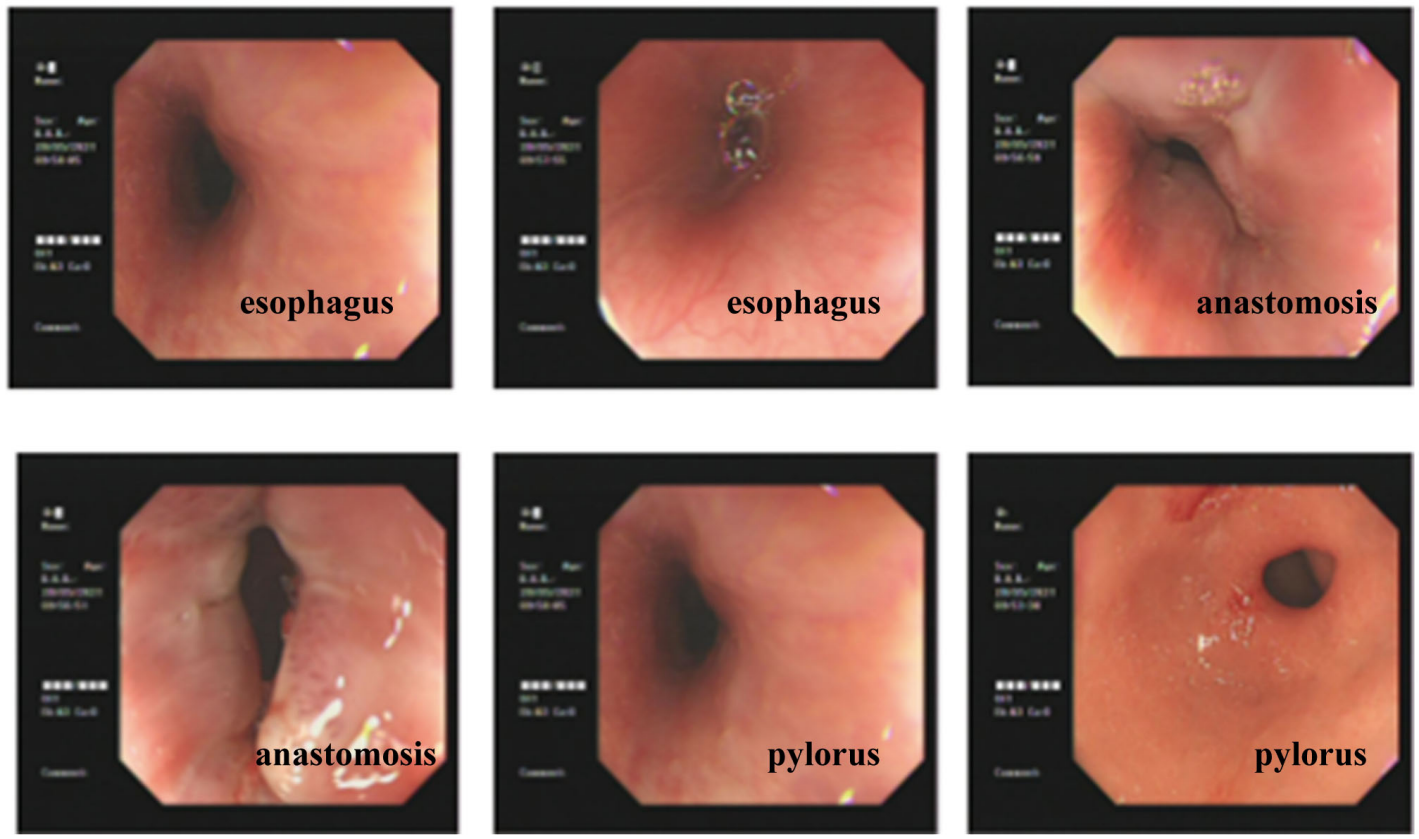
Postoperative gastroscopy of the patient showed smooth esophageal mucosa and unobstructed anastomosis.

All patients were followed up from 1 to 42 months, with a median follow-up of 24 months. None of the patients had tumor recurrence or metastasis. Laparoscopic surgery was performed on one case of anastomotic stenosis and one case of reflux esophagitis (**Figure 5**).

## 4 Discussion

At present, the main treatment for early gastric cancer is endoscopic resection. In 2002, Hashizume and Sugimachi (14) first reported the Da Vinci robotic surgical system assisted gastrectomy. The application of robotic technology in gastric cancer surgery has good safety, and can obtain near and long-term curative effects comparable to laparoscopic and open surgery (15–18). In the early stage of robotic surgery, digestive tract reconstruction after gastric cancer resection is mostly assisted by small incisions. With the iteration of anastomosis instruments and the improvement of anastomosis technology, the reconstruction of digestive tract after robotic gastrectomy has begun to move towards the era of complete endoscopic anastomosis. Advantages of complete endoscopic digestive tract reconstruction by robot: For special patients with obesity, barrel chest or high esophageal disconnection plane, small incision assisted digestive tract reconstruction has high technical requirements, limited field of vision, and difficult operation. Sometimes, to perform a safe anastomosis, the incision must be widened, resulting in the final incision length being no different from open surgery, greatly reducing the minimally invasive importance of robotic surgery. Complete endoscopic digestive tract reconstruction makes full use of the advantages of the robotic system, such as the multi-degree of freedom rotatable wrist device, high-definition enlarged 3D field of view and filtered hand tremor, and is easy to suture, which greatly reduces the difficulty of complete endoscopic digestive tract reconstruction (19). Complete endoscopic digestive tract reconstruction can narrow the incision, shorten the time of abdominal exposure, reduce the loss of abdominal fluid, and reduce the interference of internal environmental homeostasis. At the same time, compared with robot-assisted gastric cancer surgery, complete robotic surgery for gastric cancer does not significantly increase the time of digestive tract reconstruction and the incidence of postoperative complications, and the long-term survival rate of the two is comparable (20).

Compared with total gastrectomy, proximal gastrectomy can both ensure postoperative nutritional function and reduce the incidence of postoperative dumping syndrome (21–23). However, due to the particularity of the anatomical site, radical gastrectomy of proximal gastric cancer is still being explored, especially the selection of digestive tract reconstruction mode is a hot research topic at present, mainly focusing on reducing postoperative complications and improving the quality of life (24–26). The anastomosis methods of radical gastrectomy for proximal gastric cancer mainly include esophagus-residual stomach anastomosis, double-channel anastomosis, jejunal interposition anastomosis, etc. (27). Esophagogastric stump anastomosis mainly includes esophagogastric anastomosis, esophagogastric tube anastomosis, vagus nerve preservation or pyloroplasty, esophagogastric anastomosis double muscle flap plasty, etc. (28, 29). Existing studies have shown that laparoscopic assisted radical gastrectomy for proximal gastric cancer not only shows satisfactory results in terms of short-term clinical efficacy such as prevention of postoperative reflux esophagitis, but also in long-term clinical outcomes such as postoperative nutritional status (body mass and skeletal muscle indexes) and quality of life (2, 30–33).Our team previously reported the technique of esophagogastric anastomosis double muscle flap plasty after complete laparoscopic proximal gastrectomy, which obtained satisfactory short-term clinical results such as anti-esophageal reflux and good nutritional status (34). Reports in the literature on the application of robot-assisted esophagogastric anastomosis plasty with double muscle flap in proximal gastrectomy have demonstrated satisfactory results in terms of short-term clinical efficacy, such as prevention of postoperative reflux esophagitis and maintenance of good nutritional status (hemoglobin) (8). The anastomosis method of double muscle flap is manual anastomosis method, which requires a lot of suture and is difficult to operate under laparoscopy. The multi-freedom rotatable wrist device of the robot makes it more convenient to reconstruct the digestive tract after gastrectomy by manual anastomosis in the abdominal cavity. The key steps of esophagogastric anastomosis double muscle flap plasty are as follows: First, an “H” shaped mark is made on the residual stomach, the upper and lower spacing is 3.5cm, the width is 2.5cm, and the distance from the top of the residual stomach is about 3 ~ 4cm. The gastric muscle layer and mucosal layer are separated along the H-shaped marking line, and the muscle flap is made. Then the posterior wall of the esophagus was fixed on the residual stomach, and the broken end of the esophagus and the residual stomach were sutured continuously. Finally, the double muscle flap around the anastomosis was wrapped and sutured. Robot-assisted esophagogastric anastomosis double muscle flap plasty is applied in proximal gastrectomy, and the “H” shaped muscle flap is made outside the auxiliary incision, which will undoubtedly increase the length of abdominal incision and abdominal exposure time. Compared with *in vitro*, the whole cavity “H” shaped muscle flap can shorten the surgical incision to the greatest extent, reduce the chance of abdominal exposure, and lay a good foundation for the rapid recovery of patients after surgery. Based on this, the author’s team tried to carry out complete robotic esophagogastric anastomosis double muscle flap plasty, and achieved good results. On the one hand, the magnification and stereoscopic action of pneumoperitoneum and robot 3D camera can be used to provide a clearer surgical field of view and more clearly display the tissue structure of gastric muscle layer, submucosa, mucosal layer, etc., so as to carry out more detailed separation. On the other hand, full robotic surgery can reduce surgical trauma, reduce postoperative pain, and speed up postoperative recovery. The experience of the author’s team is: when making the “H” shaped muscle flap under the full robot, the assistant can pull the muscle flap vertically downwards to form a certain tension, and the surgeon uses unipolar scissors to separate the submucosa to ensure the integrity of the mucosal layer. Gradually dissociate from the middle to both sides of the “H” shape. It should be noted that the “H” shape should be made 3 ~ 4cm below the proximal end of the residual stomach and close to the greater curved side of the stomach to ensure the blood supply of the sarcomuscular flap. In this study, no ischemic changes occurred in the serosal muscle flap in 3 patients after surgery. When the pulp muscle flap covers the anastomosis, the meeting point should be located at l cm below the anastomosis. Considering the tightness of the pulp muscle flap, a “Y” shape suture should be performed to suture the top of the pulp muscle flap, without the need for the suture of the pulp muscle flap, so as to reduce the occurrence of anastomosis stenosis. The results of this study showed that 35 patients successfully completed the total laparoscopic, robotic proximal radical gastrectomy - esophagogastric anastomosis double muscle flap plasty, and all the indicators during, after and short-term follow-up were good, and the patients obtained good serum hemoglobin and nutritional status. The time of esophagogastric anastomosis, the amount of blood loss and the number of lymph nodes were significantly different between the two groups (P<0.05). This study involved only a small number of cases and further high-quality prospective multicenter randomized controlled studies are needed to confirm its long-term efficacy.

In summary, complete Da Vinci robot and laparoscopic esophagogastric anastomosis double muscle flap plasty for radical resection of proximal gastric cancer can minimize surgical incision, reduce abdominal exposure, accelerate postoperative rehabilitation of patients, and effectively prevent reflux esophagitis and maintain good nutritional status. Robotic surgery has advantages in terms of esophagogastric anastomosis double muscle flap plasty time, blood loss and lymph node dissection.

## Data availability statement

The original contributions presented in the study are included in the article/supplementary material. Further inquiries can be directed to the corresponding author.

## Ethics statement

The studies involving humans were approved by Liaoning Cancer Hospital &Institute(20210128X). The studies were conducted in accordance with the local legislation and institutional requirements. Written informed consent for participation was not required from the participants or the participants’ legal guardians/next of kin in accordance with the national legislation and institutional requirements.

## Author contributions

DY: Writing – original draft. YL: Writing – original draft. XM: Writing – original draft. XX: Writing – original draft. CW: Writing – original draft. MZ: Writing – original draft. TZ: Writing – original draft.
